# Supplementary material for: Explainable machine learning to identify chronic lymphocytic leukemia and medication use based on gut microbiome data
Source: Microbiol Spectr. 2025 Nov 6;13(12):e00944-25. doi: 10.1128/spectrum.00944-25 (PMC12671114; doi:10.1128/spectrum.00944-25)
Supplement: Supplemental Material — Supplemental methods; Fig. S1 and S2. [file spectrum.00944-25-s0001.pdf]

# Supplementary Methods

## Sample collection, sequencing and pre-processing

As previously described (1–3), fecal samples from Rigshospitalet were collected using OMNIgene.GUT stabilization tube according to the manufacturer's instructions by either the patients or the nursing staff. Briefly, samples were immediately fixated by the stabilization fluid of the OMNIgene.GUT tube, delivered to the PERSIMUNE biobank, and subsequently frozen within 72 h. All samples were stored at −80°C until shipment for sequencing. One stool aliquot for each sample was sent on dry ice for DNA extraction. Isolated DNA was sequenced in Badalona, Spain in 2017 and 2018, in South Korea (Macrogen) in 2020, and in Copenhagen (Clinical Microbiomics) in 2021 and 2023. In 2018, batches were sequenced in three separate runs. Potential batch effect in microbial profiles in individual cohorts was checked using PERMANOVA. All samples, including samples from healthy cohorts, underwent shotgun metagenomic sequencing on the Illumina Hi-Seq platform.

After sequencing, reads underwent preprocessing and quality control steps, including trimming of reads, removal of reads below 45 base pairs, and removal of all reads mapping to the human genome (hg19). Reads with a minimum identity of 80 across 90 bases were discarded from further analysis.

## Data sources and medication records

The information on medication usage in the CLL cohort was obtained from a review of electronic health records (EHR) collected from the Danish Health Data Authority (SDS) (4), and the EPIC®-based EHR system in eastern Denmark (Sundhedsplatformen [SP]), now gathered as a comprehensive data resource for Danish Lymphoid Cancer Research (DALY-CARE) approved for epidemiological, molecular, and data-driven research (5). Medication data for all other cohorts were extracted from the PERSIMUNE data warehouse, which is collected through regional and nationwide electronic data repositories. Medication data are retrieved through the Register of Pharmaceutical Statistics (LSR) (6) and SP. By including data from SP, LSR and SDS, the medication records in this project cover medicine administered and prescribed at the hospital, as well as medicine prescribed outside the hospital. We collected medication data prescribed or administered to an individual between 30 days and ≤ 1 day prior to the sampling date. Only medication used by more than 5 individuals across assessed cohorts was considered. The sampling date was missing for 12 samples and the arrival date to the biobank was used as a proxy for sampling date in these cases. Data on antineoplastic treatment was collected for patients from CLL, pre-aHSCT AML and other pre-aHSCT cohorts between 180 and ≤1 day prior to microbiome sampling.

**Taxonomical and functional profiling**

Taxonomic profiling and estimation of the relative abundances at all taxonomical levels were done using MetaPhlAn4 (7). As previously described (2,3,8), metabolic function abundances were assessed through profiling of gut metabolic modules (GMMs). In short, gene profiling was performed by mapping sequences to the integrated gene catalogue (IGC) (9) using BWA-MEM2 (10) and counting the reads that mapped to each gene, with a minimum match size of 60 and a minimum identity of 90. GMM profiling involved normalizing the length of IGC counts and length normalized counts were subsequently normalized via 16sRNA genes by dividing the sum of length normalized 16S rRNA sequences (11). The normalized counts were aggregated for each KEGG gene ontology term (12) sourced from the IGC catalog (9), and converted into GMM profiles using omixer-RPM (13). The resulting GMM profiles reflect the predicted functional potential of the microbiome and will therefore be referred to as "GMM functional profiles" throughout this work.

**Bioinformatics and statistical analyses**

To understand the variability and commonality in gut microbiome structures across different cohorts, the enterotype identification was done following the enterotyping tutorial published along with the original article by Arumugam, et al. (14). Jensen-Shannon divergence (JSD) based on relative genus abundances was calculated between pairs of samples to create a distance matrix. Partitioning Around Medoids (PAM) clustering was performed on the distance matrix to identify clusters of samples. The optimal number of clusters was determined using the Calinski-Harabasz (CH) index, and the clustering results were visualized using Principal Coordinates Analysis (PCoA). All analyses were conducted using R with the *HelpersMG*, *cluster*, *clusterSim*, and *ade4* packages.

The principal component analysis (PCA) was calculated using the *prcomp* R function with auto-scaled square root transformed relative abundance of bacterial species as input. Subsampling of 30 samples per cohort was applied. The use of square root transformation was used to alleviate the rare species bias. Once the PCA was conducted, the resulting principal components (PCs) were analyzed. Specific PCs that could explain differences between CLL patients and non-CLL patients were identified using univariate generalized linear models (GLM). The PCs with GLM $P < .05$ were selected as significant at explaining the binomial response (1: CLL; 0: non-CLL). To compare the gut microbiome structures across patient cohorts, cosine similarity was used as a metric. Cosine similarity is a measure of similarity between two vectors based on the cosine of the angle between them. To calculate angles, the centroids of each cohort were calculated as mean of the significant PC scores within the subsample of 30 samples. The angle between the centroids of two different cohorts was calculated, given centroids *a* & *b*:

$$cos\theta = \frac{a \cdot b}{\parallel a \parallel \parallel b \parallel}$$

From the cosine similarities between the cohort centroids obtained from 500 iterations, we took the mean of the cosine similarities to derive angular distances and standard deviation reported in the results section. Low cosine similarity indicates that the gut microbiome compositions are very different between the cohorts, whereas high cosine similarity suggests that the microbiome structures are similar. This approach allows for a robust comparison of the cohort structures, using the strengths of PCA (dimensionality reduction while capturing the variance) and cosine similarity, as a measure of similarity in multidimensional space.

To assess the predictive power of different metadata and medication groups on gut microbiome variance, we used redundancy analysis with the *rda* and *ordiR2step* functions from the *vegan* package. To identify associations between individual medication groups and microbial abundances and GMM functional profiles, multivariate regression analysis was used. The analysis involved a two-step regression analysis using the glm2 function as described by Nagata et al (15). Univariate regression was first run on the clr-transformed relative abundances of bacterial species and log10-transformed GMM functional profiles against medication groups to identify significant associations. Multivariate regression analysis was then applied to control for confounding factors such as age and sex, and included all significant explanatory variables identified by univariate regression. No additional corrections for multiple testing were done as only variables with BH adjusted p-values < .05 in the univariate regression analysis were included in the multivariate regression analysis.

**Machine learning models**

To perform machine learning, we used medical artificial intelligence toolbox (MAIT), a pipeline tailored for tabular data and binary classification (16). We performed 5-fold cross-validation on seven models to classify CLL as an outcome. The models included Logistic Regression, Random Forest, Naïve Bayes, Histogram-based Gradient Boosting Classification Tree (HistGBC), QLattice, LightGBM, and CatBoost. Hyperparameters were optimized using random search, with tuning conducted via 10 repetitions within each of the 5-folds to maximize classification performance. Feature selection was conducted using the minimum redundancy maximum relevance (mRMR) algorithm (17) to identify key microbial taxa and GMM functional profiles contributing to the classifications. To ensure a robust evaluation and to mitigate the risk of biased assessment associated with a single metric, the models in each cross-validation fold were evaluated by multiple performance metrics. The metrics included: Positive Predictive Value (PPV), Negative Predictive Value (NPV), sensitivity, specificity, balanced accuracy, Matthew's Correlation Coefficient (MCC), Receiver Operating Characteristic Area Under the Curve (ROC-AUC), Precision-Recall Area Under the Curve (PRAUC), Brier Score, and F1 Score. Considering that our study dealt mostly with imbalanced outcomes (positive instances < negative instances), we, among other measures, focused on MCC. MCC provides a balanced measure of the quality of classifications, considering both correct and incorrect predictions in relation to the distribution of

classes in the dataset. Correlation of the features to the outcome variable was assessed by Point-Biserial correlation with 1000 bootstrap (resampling with replacement) samples.

## References


1. Ilett EE, Jørgensen M, Noguera-Julian M, Nørgaard JC, Daugaard G, Helleberg M, et al. Associations of the gut microbiome and clinical factors with acute GVHD in allogeneic HSCT recipients. Blood Adv. 2020 Nov 24;4(22):5797–809. Available from: https://doi.org/10.1182/bloodadvances.2020002677

2. Faitová T, Svanberg R, da Cunha-Bang C, Ilett EE, Jørgensen M, Noguera-Julian M, et al. The gut microbiome in patients with chronic lymphocytic leukemia. Haematologica. 2022;107(9):2238–43. Available from: https://haematologica.org/article/view/haematol.2021.280455

3. Faitova T, Coelho M, da Cunha-Bang C, Ozturk S, Kartal E, Bork P, et al. The diversity of the microbiome impacts chronic lymphocytic leukemia development in mice and humans. Haematologica. 2024 Sep 10; Available from: https://haematologica.org/article/view/haematol.2023.284693

4. SDS. Sundhedsdatastyrelsen: The national hospital medication register. https://sundhedsdatastyrelsen.dk/da/registre-og-services/om-de-nationale-sundhedsregistre/sygdomme-laegemidler-og-behandlinger/sygehusmedicinregisteret. 2023.

5. Brieghel C, Werling M, Frederiksen CM, Parviz M, Cunha-Bang C da, Faitova T, et al. The Danish Lymphoid Cancer Research (DALY-CARE) data resource: the basis for developing data-driven hematology. medRxiv . 2024 Jan 1;2024.04.11.24305663. Available from: http://medrxiv.org/content/early/2024/04/12/2024.04.11.24305663.abstract

6. Johannesdottir SA, Horváth-Puhó E, Ehrenstein V, Schmidt M, Pedersen L, Sørensen HT. Existing data sources for clinical epidemiology: The Danish National Database of Reimbursed Prescriptions. Clin Epidemiol. 2012;4:303–13. Available from: http://dx.doi.org/10.2147/CLEP.S37587

7. Blanco-Míguez A, Beghini F, Cumbo F, McIver LJ, Thompson KN, Zolfo M, et al. Extending and improving metagenomic taxonomic profiling with uncharacterized species using MetaPhlAn 4. Nat Biotechnol . 2023;41(11):1633–44. Available from: https://doi.org/10.1038/s41587-023-01688-w

8. Jørgensen M, Nørgaard JC, Ilett EE, Marandi RZ, Noguera-Julian M, Paredes R, et al. Metabolic Potential of the Gut Microbiome Is Significantly Impacted by Conditioning Regimen in Allogeneic Hematopoietic Stem Cell Transplantation Recipients. Int J Mol Sci. 2022;23(19). Available from: http://dx.doi.org/10.3390/ijms231911115

9. Li J, Jia H, Cai X, Zhong H, Feng Q, Sunagawa S, et al. An integrated catalog of reference genes in the human gut microbiome. Nat Biotechnol. 2014;32(8):834–41. Available from: https://doi.org/10.1038/nbt.2942

10. Li H, Durbin R. Fast and accurate short read alignment with Burrows-Wheeler transform. Bioinformatics. 2009 Jul;25(14):1754–60.

11. Coelho LP, Alves R, Monteiro P, Huerta-Cepas J, Freitas AT, Bork P. NG-meta-profiler: fast processing of metagenomes using NGLess, a domain-specific language. Microbiome. 2019;7(1):84. Available from: https://doi.org/10.1186/s40168-019-0684-8

12. Kanehisa M, Goto S. KEGG: Kyoto Encyclopedia of Genes and Genomes. Nucleic Acids Res. 2000 Jan 1;28(1):27–30. Available from: https://doi.org/10.1093/nar/28.1.27

13. Darzi Y, Falony G, Vieira-Silva S, Raes J. Towards biome-specific analysis of meta-omics data. ISME J . 2016;10(5):1025–8. Available from: https://doi.org/10.1038/ismej.2015.188

14. Arumugam M, Raes J, Pelletier E, Le Paslier D, Yamada T, Mende DR, et al. Enterotypes of the human gut microbiome. Nature. 2011;473(7346):174–80. Available from: https://doi.org/10.1038/nature09944

15. Nagata N, Nishijima S, Miyoshi-Akiyama T, Kojima Y, Kimura M, Aoki R, et al. Population-level Metagenomics Uncovers Distinct Effects of Multiple Medications on the Human Gut Microbiome. Gastroenterology. 2022 Oct 1;163(4):1038–52. Available from: https://doi.org/10.1053/j.gastro.2022.06.070

16. Zargari Marandi, R., Murray, D., Milojevic M. GitHub repository. GitHub; 2024. MAIT - medical artificial intelligence. Available from: https://github.com/PERSIMUNE/MAIT

17. Ding C, Peng H. Minimum redundancy feature selection from microarray gene expression data. J Bioinform Comput Biol. 2005 Apr;3(2):185–205.

# Supplemental Figure 1

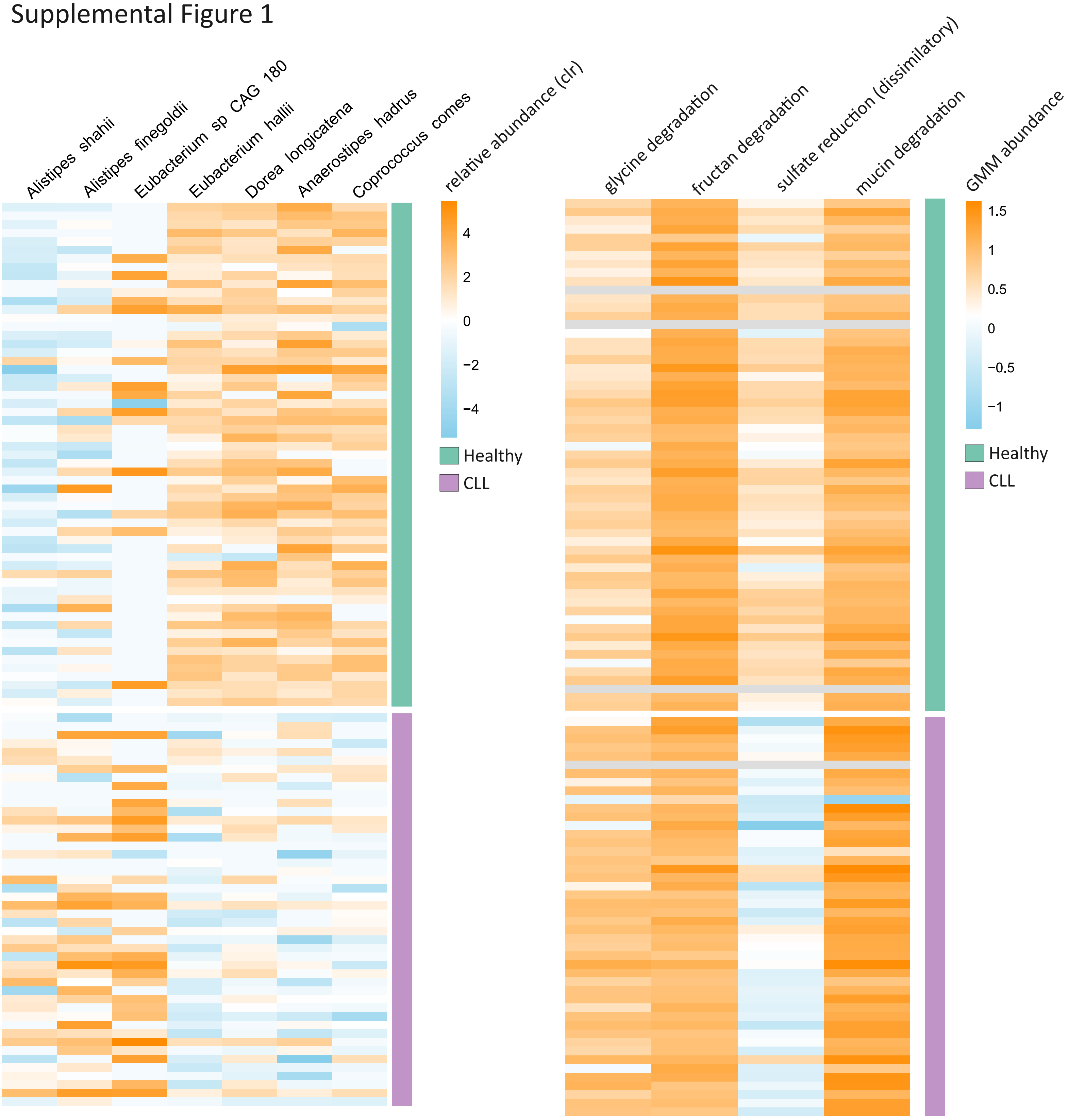


**Supplemental Figure 1.** Heatmap of relative abundance (center-log-transformed) of top 10 features used by CatBoost model for classification of CLL. CLL patients (n = 46; purple) included in this heatmap did not use any medicaiton listed in Table 2 in 30 days prior to microbiome sampling neither were not exposed to chemotherapy in 180 days prior to sampling. Healthy cohort (n = 59, green).

Supplemental Figure 2

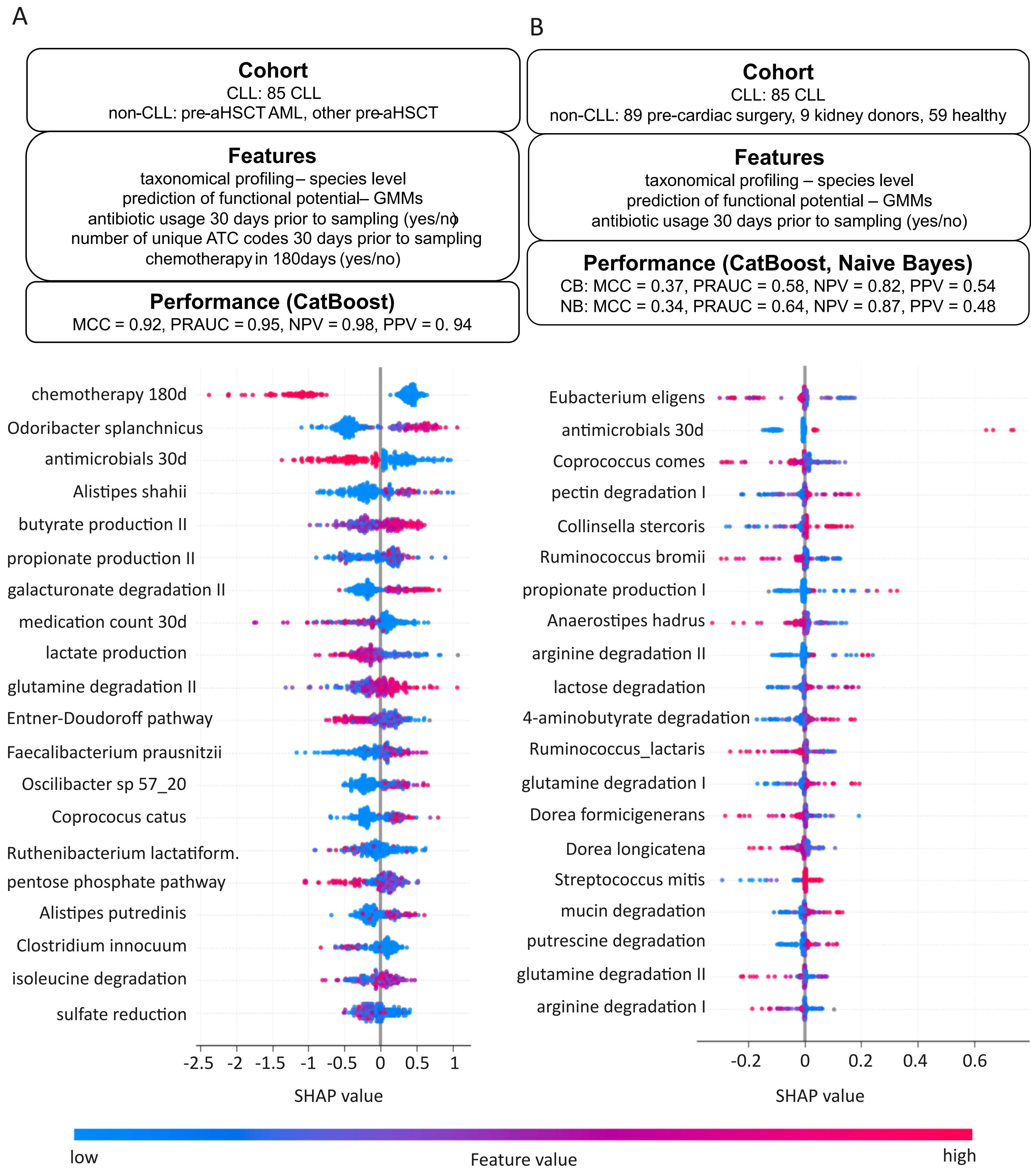

**Supplemental Figure 2. Machine learning (ML) models performance and outcome.** Classification model A) was designed to classify CLL as a binary outcome among CLL and pre-aHSCT AML and other pre-aHSCT cohorts, model B) was designed to classify CLL as a binary outcome among CLL and pre-cardiac surgery, kidney donors and healthy cohorts. Performance summarizes the performance of the best performing model by taking the average of metrics across 5-folds. Metrics used for selection of the best performing classifiers are listed. SHapley Additive exPlanation (SHAP) summary plot illustrates the top 20 contributing bacterial species, GMM functional profiles and other variables used for classification. Each dot represents one patient and is colored such that red and blue represent higher and lower attribute value of a feature, respectively. Negative SHAP values associate with decreased predicted probabilities of CLL and vice versa. MCC, Matthews Correlation Coefficient (mean value across the 5-fold validation; MCC value ranges from -1 to 1, 1 = perfect classification, 0 = random prediction); PRAUC, Precision-Recall Area Under Curve (summarizes trade-off between precision [positive predictive value] and recall [sensitivity]); PPV, Positive Predictive Value (proportion of positive instances that were correctly classified); NPV, Negative Predictive Value (proportion of negative instances that were correctly classified).
